# Brain 18 F-FDG PET reveals cortico-subcortical hypermetabolic dysfunction in juvenile neuropsychiatric systemic lupus erythematosus

**DOI:** 10.1186/s13550-024-01088-4

**Published:** 2024-04-02

**Authors:** Sebastian Rodrigo, Stefania Costi, Pierre Ellul, Melodie Aubart, Nathalie Boddaert, Stephane Auvin, Monique Elmaleh, Alexandra Ntorkou, Brigitte Bader-Meunier, Vincent Lebon, Isabelle Melki, Catherine Chiron

**Affiliations:** 1 CEA, SHFJ (Frederic Joliot Hospital), Orsay, France; 2 Biomedical Multimodal Imaging (BioMaps) Laboratory, CEA, INSERM, CNRS, and Paris-Saclay University, Orsay, France; 3 Pediatric Rheumatology Unit, ASST-PINI-CTO (Regional Health Care and Social Agency Gaetano Pini), Milan, Italy; 4 Child and Adolescent Psychiatry, APHP, Robert Debré Hospital, Paris-Cité University, Paris, France; 5 Immunology-Immunopathology-Immunotherapy (i3) Laboratory, INSERM UMR-S 959 and Sorbonne University, Paris, France; 6 Pediatric Neurology, APHP, Hospital Necker for Sick Children, Paris-Cité University, Paris, France; 7 INSERM U1163, Imagine Institute, Paris, France; 8 Pediatric Radiology, APHP, Hospital Necker for Sick Children, Paris-Cité University, Paris, France; 9 Pediatric Neurology, APHP, Robert Debré Hospital, Paris-Cité University, Institut Universitaire de France (IUF), Paris, France; 10 Pediatric Radiology, APHP, Robert Debré Hospital, Paris-Cité University, Paris, France; 11 INSERM U1141 Neurodiderot and Neurospin Institute, Paris, France; 12 Pediatric Immunology and Rhumatology, APHP, Hospital Necker for Sick Children, Paris, France; 13 Robert Debré Hospital, General Pediatrics, Infectious Disease and Internal Medicine Department, Reference center for Rheumatic, APHP, AutoImmune and Systemic diseases in children (RAISE), Paris, France; 14 Paediatrics, Rheumatology and Paediatric Internal Medicine, Children’s Hospital, Bordeaux, France; 15 Service Hospitalier Frederic Joliot (INSERM U1141), 4 Place du General Leclerc, Orsay, 91400 France

**Keywords:** Fluorodeoxyglucose, Positron emission tomography, Juvenile systemic lupus erythematosus, Neuropsychiatric systemic lupus erythematosus, Children, Statistical parametric mapping, Hypermetabolism

## Abstract

**Background:**

In juvenile systemic lupus erythematosus (j-SLE) with neuropsychiatric (NP) symptoms, there is a lack of diagnostic biomarkers. Thus, we study whether PET-FDG may identify any metabolic dysfunction in j-NPSLE.

**Methods:**

A total of 19 ^18^FDG-PET exams were consecutively performed using PET-MRI system in 11 non-sedated patients presenting with j-NPSLE (11-18y) for less than 18 months (m) and without any significant lesion at MRI. Psychiatric symptoms were scored from 0 (none) to 3 (severe) at PET time. PET images were visually analyzed and voxel-based analyses of cerebral glucose metabolism were performed using statistical parametric mapping (spm) with an age-matched control group, at threshold set > 50 voxels using both *p* < 0.001 uncorrected (unc.) and *p* < 0.05 corrected family wise error (FWE).

**Results:**

Patients exhibited mainly psychiatric symptoms, with diffuse inflammatory j-NPSLE. First PET (*n* = 11) was performed at a mean of 15y of age, second/third PET (*n* = 7/*n* = 1) 6 to 19 m later. PET individual analysis detected focal bilateral anomalies in 13/19 exams visually but 19/19 using spm (unc.), mostly hypermetabolic areas (18/19). A total of 15% of hypermetabolic areas identified by spm had been missed visually. PET group analysis (*n* = 19) did not identify any hypometabolic area, but a large bilateral cortico-subcortical hypermetabolic pattern including, by statistical decreasing order (unc.), thalamus, subthalamic brainstem, cerebellum (vermis and cortex), basal ganglia, visual, temporal and frontal cortices. Mostly the subcortical hypermetabolism survived to FWE analysis, being most intense and extensive (51% of total volume) in thalamus and subthalamus brainstem. Hypermetabolism was strictly subcortical in the most severe NP subgroup (*n* = 8, scores 2–3) whereas it also extended to cerebral cortex, mostly visual, in the less severe subgroup (*n* = 11, scores 0–1), but difference was not significant. Longitudinal visual analysis was inconclusive due to clinical heterogeneity.

**Conclusions:**

j-NPSLE patients showed a robust bilateral cortico-subcortical hypermetabolic network, focused subcortically, particularly in thalamus, proportionally to psychiatric features severity. Further studies with larger, but homogeneous, cohorts are needed to determine the sensitivity and specificity of this dysfunctional pattern as a potential biomarker in diffuse inflammatory j-NPSLE with normal brain MRI.

**Supplementary Information:**

The online version contains supplementary material available at 10.1186/s13550-024-01088-4.

## Background

Juvenile systemic lupus erythematosus (j-SLE) is a rare pediatric chronic auto-immune disease (prevalence rate: 3.76/100 000 in France in 2010) affecting multiple organs including brain leading to possible neuropsychiatric (NP) features [1]. Juvenile NP SLE (j-NPSLE) occurs in more than 20% j-SLE, respectively inducing a tenfold to threefold increase in mortality rate [2, 3]. Morbidity is also important, thus impacting considerably quality of life [4]. Although NPSLE classification diagnosis criteria and scores have been developed, attributing NP features to SLE often remains a challenge, especially in patients with mainly psychiatric symptoms, which may have implications for the therapeutic management of these manifestations [5]. No specific NPSLE biomarker has been validated so far, even if interferon and neopterins in CSF were recently reported as promising biological targets [3, 6].

Regarding brain imaging, conventional MRI is negative, or abnormalities are non-specific in more than 50% of NPSLE patients [7]. FDG-PET could be a more informative technique, as it may explore the two principal pathogenic processes involved in NPSLE: ischemia and inflammation [3]. Schematically, the former results in hypometabolism, the latter in hypermetabolism [8, 9]. More generally, both processes are involved in the wide field of the autoimmune encephalitis (AE), of which NPSLE is one of many subtypes. Large series of patients with AE, both adults and children, underwent PET-FDG examination: a mixed pattern of hypo- and hypermetabolism is currently considered to support the diagnosis of AE [10–12]. However, the specific FDG-PET characteristics of the different types of AE within this heterogeneous generic group have not been addressed, especially for SLE.

Most FDG-PET studies dedicated to NPSLE have involved adult patients and focused on hypometabolism without investigating any potential hypermetabolism [13–16]. More recently, hypermetabolic areas were reported in anecdotical pediatric cases [10, 17, 18]. But, the purely visual image analysis used in these studies, or based on predetermined regions of interest, leads to substantial bias in the detection and localization of metabolic anomalies. The same applies to the previously mentioned FDG-PET studies in AE.

We report here the first pediatric series of NPSLE patients explored by FDG-PET. To minimize analysis bias, cases with significant ischemic lesions were excluded and patients were studied on a PET/MR scanner using whole brain voxel-based method against a previously published FDG-PET data base of pediatric controls [19].

## Methods

This is a monocentric retrospective study on an exhaustive series of consecutive patients. The study was approved by our institutional review board and the requirement to obtain informed consent was waived.

### Patients

Inclusion criteria were the following: (i) SLE diagnosis based on the 2019 American College of Rheumatology (ACR)/ European Alliance of Associations for Rheumatology (EULAR) criteria (ii) SLE onset before 16 years of age (j-SLE) (iii) Patient followed in the Reference center for Rheumatic, AutoImmune and Systemic diseases in children (RAISE) and assessed in the Excellence Center for Neuro-developmental Disorders (InovAND) (iv) with NP features, occurring before the age of 18 years and attributed to SLE (j-NPSLE), (v) brain MRI previously performed (vi) PET examination feasible without sedation. Patients with extended cortical infarcts on MRI (involving at least a complete lobe) were excluded. Some patients were longitudinally assessed after first FDG-PET evaluation.

NP symptoms were attributed to SLE after clinical assessment by the multidisciplinary team of RAISE and InovAND (pediatric neurologist, psychiatrist, rheumatologist and psychologist) according to NPSLE 1999 ACR criteria. Several scores were recorded at NPSLE diagnosis and at time of PET: SLE Disease Activity Index (SLEDAI) and a local clinical NP score of 0 (none), 1 (mild), 2 (moderate), 3 (severe) for each of the ten main NP symptoms in SLE (mood disorders, depression, anxiety, inversion of the nocturnal rhythm, visual/auditive hallucinations, catatonia, acute confusion, cognition disorders, psychomotor slowing, acute confusion).

### PET imaging

PET was performed using a 3 T PET-MRI scanner (Signa PET/MR, GE Healthcare, Waukesha, WI, USA), except in 3 exams for whom we used an ECAT HRRT Siemens PET scanner because of an unremovable dental device. Patients fasted for a minimum of 4 h prior to PET. They received intravenous injection of 3.7 MBq of ^18^F-FDG and were isolated in a dedicated room during the uptake period in the presence of their family with the instruction to rest with minimal auditory and visual stimulation. No subject received sedation for the purpose of PET. After 30 min the subject was installed in the PET-MRI system for 15 min of brain image acquisition.

### PET individual analysis

First, visual analysis was performed to identify and localize both hypometabolic and hypermetabolic brain areas. Images were independently reviewed by three nuclear medicine specialists (SR, VL, CC) who were not blind from clinical data. Their consensus analysis is reported here.

Second, SPM analysis was performed comparing each individual PET exam to our pediatric database of 24 pseudo-controls aged 5 to 18 years, previously examined using ECAT HR + with the same ^18^F-FDG dose and acquisition procedures as for PET-MRI [19]. It was achieved using MATLAB2016 (Mathworks Inc., Natick, MA, USA) and the SPM12 (www.fil.ion.ucl.ac.uk). Each subject was spatially normalized using a specific pediatric FDG template previously built from the control group using affine and nonlinear registration algorithm by means of SPM and then smoothed (gaussian kernel, 8 mm full width at half maximum). We compared each individual against controls using a 2-sample t test, age as covariate. Both hypometabolic and hypermetabolic areas were detected using uncorrected *p* < 0.001 and > 50 voxels extent threshold. From each SPM map we extracted from all significant sets of voxels (clusters) the volume (cluster volume) and the mean statistical value (cluster mean Z-score) using the following set of regions of interest (ROI): whole brain, hemispheric cortical ribbon, subcortical areas, i.e. striatum (caudate & pallidum), putamen, thalamus, subthalamic brainstem, cerebellar hemispheres and cerebellar vermis. We then proceeded correlation tests between each individual cluster volume or cluster mean Z-score against individual NP global score. The ROIs were extracted from AAL (Automated Anatomical Labeling) [20].

### PET group analyses

SPM analyses were performed by means of full factorial analysis, age as covariate to estimate any age effect, in order to capture disease-related differences when comparing each of the following patient groups to the pseudo-control group mentioned above: all PET exams (*n* = 19), first PET exams (*n* = 11), second/third PET exams (*n* = 8), PET with minor NP disorders (all scores ≤ 1) (*n* = 11), PET with major NP disorders (at least one score ≥ 2) (*n* = 8). In addition to the same uncorrected thresholds as in individual analysis, we used family wise error (FWE) corrected *p* < 0.05 and 50 voxels extent threshold. Additionally, we proceeded to the same analysis, adding NP scores as covariate.

## Results

### Patients (Table 1)

**Table 1:** Clinical, MRI and therapeutic data of the cohort of j-NPSLE patients

Patient	Time	Age	MRI	Ttt	SLEDAI	Neurological features	Mood score	Dep score	Anx score	Rhyt score	vHal score	aHal score	Cat score	Cog score	Slow score	Conf score	Global NP score
#1	Dg PET1	16y9m 18y3m	Sc,Inf	GLpo/MMF	13 8	- -	2 0	3 0	2 2	0 0	0 0	0 0	0 0	2 0	2 0	0 0	11 2
#2	Dg PET1	14y 14y7m	Sc	Gpo/MMF/H	42 0	- -	0 1	0 0	0 0	2 1	2 0	2 0	0 0	2 1	2 0	0 0	10 3
#3	Dg PET1	11y7m 11y8m	Nl	Gpo/CYC/H	26 0	- -	0 0	0 0	0 0	2 0	0 0	2 0	0 0	3 0	0 0	0 0	7 0
#4	Dg PET1	15y3m 15y11m	Sc	Gpo/MMF/H	24 18	- -	1 1	1 1	0 0	1 0	2 0	1 0	0 0	1 1	0 0	0 0	7 3
#5	Dg PET1 PET2	15y4m 15y6m 16y	Sc Sc	Gpo/CYC Gpo	36 36 NA	Pyr SD, Tetraplegia Distal motor deficit -	0 0 0	0 0 0	0 0 0	3 0 0	2 0 0	1 0 0	0 0 0	3 1 0	2 0 0	0 0 0	11 1 0
#6	Dg PET1 PET2	16y4m 16y4m 17y9m	Nl Nl	Gpo/H Gpo/MMF/H	8 8 8	- - -	3 3 2	3 3 2	2 2 3	0 0 2	2 2 0	2 2 0	0 0 0	1 1 0	2 2 0	0 0 0	15 15 9
#7	Dg (CT)PET1 PET2	15y4m 16y1m 16y10m	Nl Nl	Gpo/MMF/H MMF/H	21 NA 20	- - -	2 0 2	2 0 2	0 0 0	2 1 2	1 0 2	0 0 2	0 0 0	2 0 0	1 0 2	0 0 0	10 1 12
#8	Dg PET1 PET2	14y 15y5m 16y2m	Nl Nl	MMF/H -	NA NA NA	- - -	0 0	0 1	0 1	0 0	0 0	0 0	0 0	0 1	0 0	0 0	0 3
#9	Dg PET1 PET2	11y3m 11y5m 12y4m	At At	Gpo/END Gpo/MMF	48 6 6	Pyr SD - -	1 0 0	0 0 0	0 0 0	0 1 0	3 0 0	3 0 0	3 1 0	3 2 1	3 0 0	3 0 0	19 4 1
#10	Dg (CT)PET1 (CT)PET2	14y11m 15y1m 15y7m	At	Gpo/CYC/H Gpo/H	23 10 16	Pyr SD - -	0 2 1	0 1 1	0 1 3	1 2 0	0 0 0	0 0 0	0 0 0	1 0 1	0 0 2	0 0 0	2 6 8
#11	Dg PET1 PET2 PET3	13y5m 14y7m 14y10m 16y5m	Nl Nl Nl	Gpo/ABA/H Gpo/ABA/H Gpo/ABA/H	22 2 2 22	- - - -	3 1 1 2	3 1 1 3	0 0 0 0	0 0 0 3	2 1 0 0	2 1 0 0	0 0 0 0	1 0 1 2	0 0 0 0	0 0 0 0	11 4 3 10

*Dg* Diagnosis of NPSLE (Neuropsychiatric lupus), *PET* Positron Emission Tomography, *y* years, *m* months, *-* none

*MRI* Nl = normal Sc = subcortical hypersignal (T2, FLAIR) At = cortico-subcortical atrophia (T1) Inf = limited cortical infarct (T1, T2, FLAIR)

*Ttt* Treatment: *Gpo* Glucocorticoide per os, *Gbo* Glucocorticoide bolus, *MMF* Mycophenolate, *CYC* Cyclophosphamide, *ABA* Abatacept, *END* Endoxan, *H* Hydroxychloroquine

*SLEDAI* SLE (Systemic Lupus Erythematosus) Disease Activity Index, *Pyr SD* Pyramidal syndrome

*Dep* Depression, *Anx* Anxiety, *Rhyt* Inversion of the nocturnal rhythm, *vHal*/*aHal* Visual/Auditory Hallucinations, *Cat* Catatonia, *Cog* Cognition, *Slow* Psychomotor slowing, *Conf* Acute mental confusion, *NP* Neuropsychiatric

*Local Neuropsychiatric (NP) scores* (including exclusively the psychiatric symptoms): 0 = none, 1 = mild, 2 = moderate, 3 = severe

Twelve pediatric patients with diagnosed NPSLE were referred for brain FDG-PET between September 2019 and July 2022. Eleven of them were prospectively included in this study, the remaining one being excluded for an infarct of the whole left temporal lobe with extended loss of substance. Mean age at diagnosis was 14y6m (SD 2y4m). NP scores are detailed in Table 1. Of note, the patients presented with central nervous system (CNS) impairment, mainly psychiatric manifestations; none of them had seizures, motor deficit, or abnormal movements at time of PET. No patient displayed peripheral nervous system (PNS) features.

PET was repeated 2 times in 6 patients and 3 times in 1 patient, so that a total of 19 PET scans were studied. Mean age was 15y (SD 2y5m) at first PET and 15y7m (SD 8 m) at second PET, with timelag between j-NPSLE diagnosis and first PET ranging from 0 to 18 m (mean 7 m, SD 3 m) and timelag between two PET exams ranging from 6 to 19 m (mean 9 m, DS 9 m). All exams but one, were performed upon treatment, which usually associated oral glucocorticoids (*n* = 15/18), immunosuppressive therapy (mycophenolate, ciclophosphamide, abatacept or endoxan) (*n* = 16/18), and hydroxychloroquine (*n* = 15/18). Treatment remained unchanged from one PET to the next, except for stopping the immunosuppressor in 2 cases and glucocorticoids in 1 case (Table 1).

Cerebral MRI at time of PET disclosed significant abnormalities in 2 patients: two limited infarct lesions in the left superior temporal gyrus and the right temporal operculum (patient #1), diffuse hypersignal in the cortex and basal ganglia (patient #5). Abnormalities were insignificant in 4 other patients (punctiform subcortical hypersignals in 2, mild to moderate cortico-subcortical atrophy in 2) and MRI was negative in the 5 remaining ones.

### PET group analysis (Table 2).

**Table 2:** SPM group analysis

SPM statistical thresholds*		*p* < 0.001 uncorrected		*p* < 0.05 FWE corrected							
PET group vs. controls		All PET exams (*n* = 19)		All PET exams (*n* = 19)		First PET exams (*n* = 11)		PET with minor NP scores (*n* = 11)		PET with major NP scores (*n* = 8)	
		Mean Z-score	Mean volume	Mean Z-score	Mean volume	Mean Z-score	Mean volume	Mean Z-score	Mean volume	Mean Z-score	Mean volume
**Whole brain**		4,24	150,248	6,20	15,232	5,94	600	6,11	7744	5,85	4832
**Cortex & hippocampus**	Occipital**	4,06	25,888	5,77	1144	0	0	5,89	1720	0	0
	Hippocampus & amygdala	3,93	6112	6	144	0	0	6	120	0	0
	Frontal lobe	3,88	8384	0	0	0	0	5,69	432	0	0
**Subcortical areas**	Striatum & putamen	4,17	8328	5,75	192	0	0	0	0	5,72	192
	Thalamus	5,40	11,064	6,56	5536	5,94	600	6,30	3752	5,83	1952
	Sub-thalamic brainstem	4,85	7064	6,37	2256	0	0	6,05	1256	5,99	672
	Cerebellar cortex	4,24	29,736	5,98	2816	0	0	5,50	16	5,89	1272
	Vermis	4,52	6632	5,56	1088	0	0	0	0	0	0

*extent threshold set to 50 voxels

**primary & associative visual cortex, *SPM* Statistical Parametric Mapping, *PET* Positron Emission Tomography, *NP* Neuropsychiatric

Minor NP score = all scores ≤ 1 (*n* = 11), Major NP scores = at least one score ≥ 2 (*n* = 8)

**Fig. 1:**
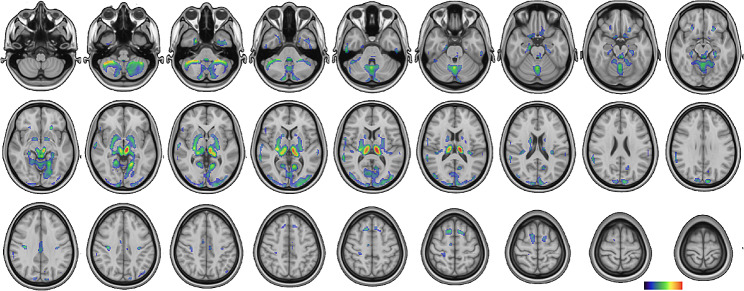
Hypermetabolic network - SPM group analysis (uncorrected). Comparing all PET exams (*n* = 19) to age-matched pseudo-controls using uncorrected *p* < 0.001 and 50 voxels threshold, significant hypermetabolism extended bilaterally through the occipital (primary and associative occipital areas), temporal and frontal cortices as well as thalamus, basal ganglia (caudate, putamen, pallidum, subthalamic brainstem) and cerebellum (vermis and hemispheres)

Comparing all PET exams (*n* = 19) to controls an extensive cortical and subcortical bilateral hypermetabolic pattern was identified, but no significant hypometabolic area emerged. Using uncorrected *p* < 0.001 and 50 voxels threshold, significant hypermetabolism extended through the occipital, temporal and frontal cortices as well as thalamus, basal ganglia (caudate, putamen, pallidum, subthalamic brainstem) and cerebellum (vermis and hemispheres) (Table 2; Fig. 1). Occipital cortex (primary and associative visual areas) and cerebellum showed the largest hypermetabolic clusters, subcortical areas the strongest ones. Using corrected FWE *p* < 0.05 the whole subcortical hypermetabolic network remained significant, but only the occipital cortex did (Table 2). Thalamus was the predominant hypermetabolic structure, showing the highest Z-score (6.56) and accounting for 36% of the whole brain hypermetabolic volume (51% together with the subthalamic brainstem), followed by cerebellum (26%), while basal ganglia (striatum & putamen) and cerebral cortex represented only 1% and 8% respectively (Table 2).

Restricting the analysis to the group of first PET exams (*n* = 11) a similar hypermetabolic pattern was identifiable using uncorrected *p* < 0.001. No significant voxels survived to FWE corrected *p* < 0.05 except the thalamus.

### PET individual analysis (Table 3)

**Table 3:** Individual analysis (visual and SPM)

Patient	Visual analysis		SPM analysis(uncorrected, p < 0.001, 50 voxels)											
	HYPER	HYPO	HYPER									HYPER	HYPO	HYPO
			Th	Mes	BG	Cer V	Cer H	Cx PV	Cx AV	Cx Au	Cx Other	Total number		Total number
#1		T_L_T_R_						_L_			FP	15	T_L_T_R_	2
#2					_R_					_R_	FP	4	P_L_	1
#3												0	PV_R_	1
#4	PV,BG										FP	20		0
#5 PET1	PV,AV,Au,Th,BG	FP,Rol									T_R_	13	FP,Rol	4
PET2	PV,AV,Au,Th,BG,Cer	FP,Rol										19		0
#6 PET1	PV,AV,Au				_R_						P	9		0
PET2	PV,AV,Au				_R_					_R_	FP	8		0
#7 PET1		F										3		0
PET2					_R_						F_R_	2		0
#8 PET1											FP	8		0
PET2	PV,Th,BG									_R_	P	9		0
#9 PET1	PV,AV,BG										FPT	15	T	2
PET2					_R_						FPT	12		0
#10 PET1	PV,AV,Th,BG,Cer											10	F	2
PET2	Th,BG,Cer											10	F	2
#11 PET1		T,Occ,Cer			_R_						FP	7		0
PET2											FP	14		0
PET3	PV,Th,BG											15		0

*HYPER* Hypermetabolism (in orange if present), *HYPO* Hypometabolism (in green if present),

* Bilateral, excepted if precised *L* left, *R* Right, or non applicable (Mesencephale or Vermis)

*Th* Thalamus, *Mes* Mesencephale, *BG* Basal ganglia, *Caud* Caudate, *Pall* Pallidum, *Put* Putamen, *Cer* Cerebellum, *V* Vermis, *H* Hemispheres, *Cx* Cortex, *PV* Primary visual, *AV* Associative visual, *Au* Auditory, *F* Frontal, *P* Parietal, *Occ* Occipital, *Op* Operculum, *T* Temporal

PET visually analyzed was abnormal in 68% of the exams (13/19), showing only hypometabolisms (3/13), only hypermetabolisms (8/13), or both (2/13). Hypometabolisms corresponded to cortical lesions on MRI in 2/4 cases. Hypermetabolisms were always reported without underlying lesions; they were mostly located in visual cortex (primary and associative), basal ganglia and thalamus (Table 3).

Using SPM analysis (uncorrected *p* < 0.001 and > 50 voxels) PET was abnormal in 100% of the exams, showing only hypermetabolisms (12/19), only hypometabolisms (1/19), or both (6/19) (Table 3). There were at maximum 2 areas of hypometabolism identified in 6 exams, mesial frontal or temporal in location; the 5 others did not disclose any. Among hypometabolisms visually detected, only the two corresponding to MRI lesions were confirmed by SPM. By contrast, there were multiple and bilateral areas of hypermetabolism identified by SPM in all PET exams but one (Table 3, Supplementary Fig. 1). A total of 15% of the hypermetabolisms identified by SPM had been visually missed, resulting in 9/19 PET exams without any hypermetabolism visually compared to only 1/19 using SPM.

### PET – relationship with disease time course and NP scores

SPM comparison between the first (*n* = 11) and second/third PETs (*n* = 8) showed no significant difference, using either individual or group analysis.

No significant correlation was found between NP scores and SPM hypermetabolic cluster values (volume or Z-score) using individual or group analysis, neither for each nor for global NP score. However, splitting NP scores into two conditions according to severity, with and without major NP symptoms (NP scores 2–3 vs. 0–1, *n* = 8 vs. *n* = 11), a different group pattern emerged using SPM group analysis (Table 2): hypermetabolism concentrated on subcortical areas in the most severe group whereas it also involved cortical areas in the less severe one (Supplementary-Figure). At corrected FWE *p* < 0.05, subcortical hypermetabolism accounted for 85% of the whole brain volume and no cluster survived on cerebral cortex in the most severe group, whereas cerebral cortex (highly preferentially the visual cortex) accounted for 29% of the whole brain volume in the less severe one (Table 2). Regardless of the NP condition, thalamus was the predominant hypermetabolic structure, both in volume and intensity. In addition, it accounted for half and two-thirds of the whole brain volume in the two NP groups respectively, when associated with subthalamic area. No other subcortical clusters remained significant in the less severe NP group; in the most severe one, cerebellum accounted for a quarter of the whole brain volume and basal ganglia for less than 5%.

**Fig. 2:**
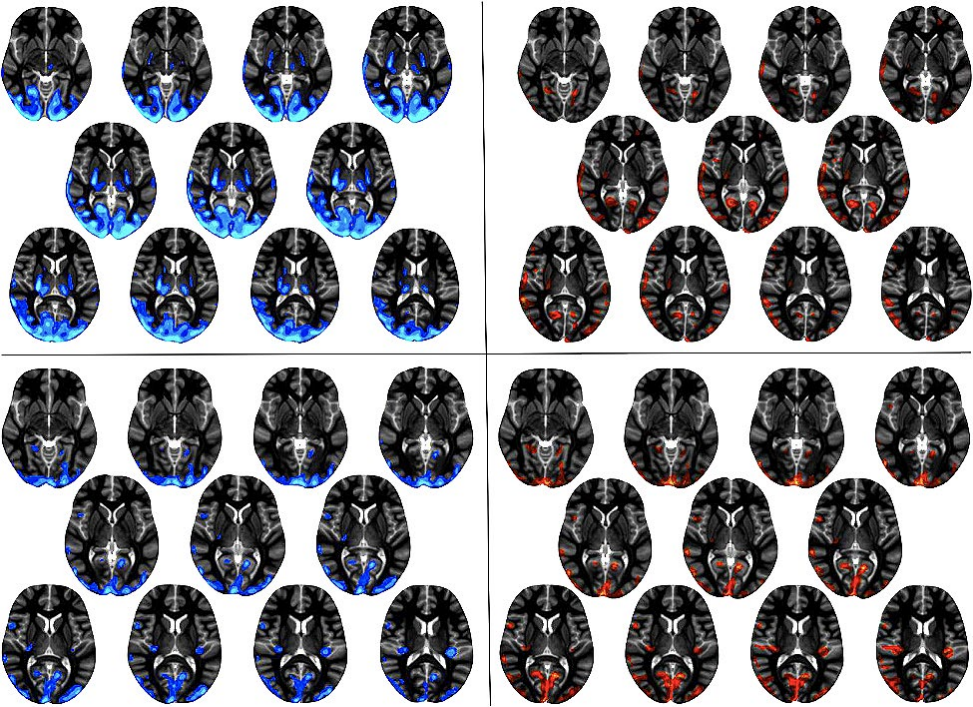
Hypermetabolism at 2 consecutive PET exams. Comparing the two consecutive PET exams using SPM individual analysis (uncorrected *p* < 0.001 and > 50 voxels) in 2 patients whose the overall neuropsychological score improved, PET1 on the left side (hypermetabolic network in blue), PET2 on the right side (hypermetabolic network in red), hypermetabolic network regressed (case #9, at the top) or remain unchanged (case #10, at the bottom)

In patients who underwent multiple PET examinations, the search for a possible correspondence between image changes and clinical condition was inconclusive, due to individual variability. For instance, while the overall NP score improved, the hypermetabolisms could regress (1 case) or remain unchanged (1 case) (Fig. 2); while the NP score worsened, the hypermetabolisms could worsen (1 case) or remain unchanged (2 cases). Similarly, the attempt to match hypermetabolisms’ location with a specific NP semiology was disappointing. For instance, visual hallucinations still present by the time of PET in 3/19 cases (they had been reported at j-NPSLE diagnosis in 7/11 patients) were associated to bilateral hypermetabolism in visual cortex in 2 of them, but a similar hypermetabolism was also found in 10 other PET examinations, without any visual hallucinations.

## Discussion

In this study we have evaluated for the first time with FDG-PET the brain dysfunction in j-NPSLE. Using a whole pediatric brain voxel-based methodology, FDG-PET group analysis identified a robust and widespread pattern of bilateral cortico- subcortical hypermetabolism, predominantly subcortical and mostly thalamic, but no hypometabolic area. Hypermetabolism was essentially subcortical in case of major NP symptoms, whereas it also extended to cerebral cortex in cases of minor NP symptoms. At the individual level, hypermetabolic abnormalities were detected in 95% of PET exams provided visual analysis was complemented by SPM analysis; otherwise, they were missed in 40% of cases. This particular metabolic profile could be of some diagnostic help, especially in patients with psychiatric symptoms but normal neurological examination and negative MRI. Of note, the patients reported in this cohort presented homogeneous j-NPSLE NP features, mainly psychiatric; none displayed neurological defect or seizures (nor PNS impairment), as it may sometimes more rarely be reported in j-NPSLE. Therefore, we do not know if such clinical signs would be associated to similar FDG patterns, and these observations would need to be evaluated on a larger cohort.

The metabolic abnormalities identified in the present study are particularly robust. A strict methodology was used to minimize the biases of most clinical PET studies in this field. Firstly, this is an exhaustive series, with all eligible patients included for analysis; it is a monocentric series, with a confirmed and relatively recent diagnosis, which reduces clinical and treatment heterogeneity; performing PET without sedation prevents the impact of drugs on metabolism; the absence of epileptic seizures and abnormal movements eliminates confounding factors for hypermetabolism, and the absence of neurological deficits and extensive/multiple clastic lesions confounders for hypometabolism. Secondly, the classic clinical visual analysis of the images, which is highly investigator-dependent and has limited sensitivity, was supplemented by an SPM analysis, which enables the detection of all the clusters significantly different from controls, without any *a priori* on pre-determined regions of interest. An age-matched pediatric database was used to avoid any bias associated with adult controls [19]. This database has proved useful in previous studies of childhood epilepsy [21–23]. In addition to the already relatively high uncorrected threshold *p* < 0.001 used in individual analysis, a more stringent corrected one was added for group analysis. Finally, the patients were explored on a PET-MR scanner, a notable benefit to optimize PET/MRI registration and thus the anatomical localization of PET anomalies.

To date, there are very few cases of j-NPSLE having experienced FDG-PET: in 3 patients, only hypermetabolism was detected, in basal ganglia using *a priori* ROI-localization [10], while in 3 others, analyzed visually, there was hypometabolism associated in cerebral cortex [17, 18]. Note that the latter had a purely psychiatric form of j-SLE, while the former exhibit solely neurological signs (seizures, ataxia). By contrast, many FDG-PET studies were carried out in adult NPSLE patients 10 to 20 years ago, but none using SPM: they all reported cortical hypometabolisms, frontal, parietal, temporal, and/or occipital in location, with a predominance on parieto-occipital, including in patients without any lesion on MRI [13–16]. It is likely that hypermetabolisms were missed at this time, partly because the methodology used for image analysis was not favorable to identify any. Hypermetabolisms had only been searched for in patients with choreiform movements (thus with a clinical hypothesis) and visually identified in basal ganglia [24].

Interest in hypermetabolism has really developed in the last few years, principally in the autoimmune encephalitis (AE). A meta-analysis on more than 700 adult patients shows an excellent sensitivity of PET (90% detection of abnormalities against 60% for MRI) with a strong diagnostic value [11]. Various metabolic patterns are reported according to the type of AE hypometabolism only (mesial temporal lobes, cerebellum, etc.), hypermetabolism only (basal ganglia, mesial temporal lobes, etc.), or both. Similar results are reported in pediatrics: in 34 children with AE, PET was retrospectively abnormal in 100% of cases, associating large cortical hypometabolisms to hypermetabolisms in 82%, the latter in basal ganglia in 59% of cases [10]; in 104 other children, 70% of whom with NP disorders, PET prospectively shows large cortical hypometabolisms with (57%) or without (36%) hypermetabolisms (especially in the basal ganglia 82%) [12]. In this last series PET sensitivity, specificity, positive predictive value and negative predictive value for AE reach 93%, 84%, 89% and 91% respectively. Based on these data, PET is now considered a diagnostic marker in AEs and hypermetabolism of basal ganglia suggestive of an autoimmune process. However, note that patients with associated clastic lesions have largely been included in these AE studies, increasing the incidence of hypometabolism, and none of them have used SPM, making the definite detection and localization of hypo- and hypermetabolisms open to discussion. Moreover, the potential differences in metabolic pattern according to the type of AE have not been formally studied. Interestingly, the 3 SLE children included in the aforementioned AE series disclosed exclusively hypermetabolisms (in basal ganglia), while hypometabolisms were missing, as in the present series [10].

Only one series has used SPM so far in SLE, but in adults and without NP features [25, 26]. They directly compared the whole group of SLE patients to healthy subjects. The pattern reported is close to ours at comparable thresholds: a largely predominant hypermetabolism, bilaterally in putamen/pallidum/thalamus, hippocampus, occipital and frontal cortex at uncorrected *p* < 0.001, surviving in hippocampi and unilaterally in putamen/pallidum/thalamus and frontal cortex at corrected *p* < 0.05. However, in our pediatric series of NPSLE, subcortical hypermetabolism is more intense (Z-score max at 6.6 vs. 4.7), it remains bilateral at corrected threshold, and it predominates in thalamus (instead of striatum in Mackay’s). It also significantly involves the subthalamic nuclei and cerebellum, which are not affected in Mackay’s study. These differences could reflect the gap in age between both studies (15 vs. 40 years in mean), particularly for the thalamus, whose metabolism is known to increase with brain maturation until around 25 years and then decrease [27]. However, age-matched control groups are similarly affected by this physiological phenomenon, thus eliminating any interference of aging process. Clinical symptomatology could therefore be responsible for the slight differences rather than age, NP symptoms having been excluded by Mackay, whereas our patients were more severely impaired, with NPSLE diagnosis confirmed. One might hypothesize a physiopathology continuum of NP features in j-SLE, as adult patients reported mood and cognitive disorders to a lesser extent than our patients.

The correspondence between hypermetabolism and NP features is a difficult issue. As opposed to hypometabolism, specific cortical patterns of which have been significantly linked to many conditions - dementia [28–30], hallucinations [31, 32], or anxiety/depression, including in children [33], hypermetabolism is much rarer. It has been found, apart from AEs, in narcolepsy [34] and Tourette’s [35]. The classical SLEDAI score in SLE was not presently optimal for correlations as it includes many non-NP components. We therefore used a NP focused scoring. A significant association between hippocampal hypermetabolism and poor spatial memory performance was identified in adult non-NPSLE patients [26]. In the present j-NPSLE study, no significant correlation was found between NP features, their type, number or severity and hypermetabolism, its localization, extension or severity. These results were expected given the limited number of patients and the heterogeneity of NP symptoms, despite a homogeneous cohort, as no patient displayed vascular or focal j-NPSLE feature. However, our group analyses suggest a metabolic spectrum, which seems to follow the intensification of NP symptoms: the cortical hypermetabolism tends to vanish behind the subcortical one and concentrate particularly in thalamus, to the detriment of the visual cortex and hippocampus. This is consistent with the fact that the thalamus plays a major role of connective hub in brain organization, even stronger than cortex does [36]. Thalamic hubs are thought to be involved in many cognitive and behavioral domains, including those involved in j-NPSLE.

Despite stringent methodology, our study retains some limitations. Small samples and symptoms heterogeneity, inherent to such a rare disease, preclude drawing definite conclusions on the course of hypermetabolism and its potential relationships with clinical features. Similarly, exclusively psychiatric patients should be compared with exclusively neurological patients, in order to gain a better understanding of their respective metabolic signatures. Exploring a control group of non-j-NPSLE patients would be the only way to determine what metabolic abnormalities are related to j-SLE pathology per se, but such a study is ethically not feasible in children.

Finally, having identified such a hypermetabolic group pattern of j-NPSLE does not mean that it can be identified in clinical practice on a given patient’s PET. Visual analysis is prone to significant interpretation bias, especially in the case of bilateral abnormalities, which prevent the perception of any asymmetry. In addition, the interpretation of an anomaly as hyper- or hypometabolism depends on the contrast scale setting. Moreover, this pattern may be variably pronounced in different individuals and associated with other anomalies. Single subject against controls SPM analysis is therefore highly recommended for the clinical interpretation of FDG-PET images in this context: in our experience, it has been beneficial in 40% of cases.

## Conclusion

These preliminary results reveal bilateral cortico-subcortical dysfunction in j-SLE with psychiatric manifestations, such as FDG-PET hypermetabolism, prominent in the thalamus. Although it is not possible at this stage to conclude on the specificity of this pattern, FDG-PET may represent an interesting imaging biomarker in the future in the context of j-NPSLE. FDG-PET as additional investigation and carefully analyzed may possibly constitute diagnostic help, especially if cerebral MRI is negative. FDG-PET also opens the way to the still unknown localization of neuronal dysfunctions underlying psychiatric features of j-NPSLE [5].

### Electronic supplementary material

Below is the link to the electronic supplementary material.

Supplementary Material 1

## Data Availability

The datasets generated and/or analysed during the current study are not publicly available due medical confidentiality but are available from the corresponding author on reasonable request.

## Abbreviations

^18^FDG: deoxyglucose labeled with 18fluor
PET: positron emission tomography
j-NPSLE: juvenile systemic lupus erythematosus with neuropsychiatric symptoms
MRI: magnetic resonance imaging
SPM: statistical parametric mapping
FWE: family wise error
SD: standard deviation

## References

[1] Arnaud L, Fagot JP, Mathian A, Paita M, Fagot-Campagna A, Amoura Z (2014). Prevalence and incidence of systemic lupus erythematosus in France: a 2010 nation-wide population-based study. Autoimmun Rev.

[2] Schwartz N, Stock AD, Putterman C (2019). Neuropsychiatric lupus: new mechanistic insights and future treatment directions. Nat Rev Rheumatol.

[3] Govoni M, Hanly JG (2020). The management of neuropsychiatric lupus in the 21st century: still so many unmet needs?. Rheumatology (Oxford).

[4] Hanly JG, Urowitz MB, Su L, Bae SC, Gordon C, Wallace DJ (2010). Prospective analysis of neuropsychiatric events in an international disease inception cohort of patients with systemic lupus erythematosus. Ann Rheum Dis.

[5] Deijns SJ, Broen JCA, Kruyt ND, Schubart CD, Andreoli L, Tincani A (2020). The immunologic etiology of psychiatric manifestations in systemic lupus erythematosus: a narrative review on the role of the blood brain barrier, antibodies, cytokines and chemokines. Autoimmun Rev.

[6] Labouret M, Costi S, Bondet V, Trebossen V, Le Roux E, Ntorkou A (2023). Juvenile neuropsychiatric systemic lupus erythematosus: identification of Novel Central Neuroinflammation biomarkers. J Clin Immunol.

[7] Luyendijk J, Steens SC, Ouwendijk WJ, Steup-Beekman GM, Bollen EL, van der Grond J (2011). Neuropsychiatric systemic lupus erythematosus: lessons learned from magnetic resonance imaging. Arthritis Rheum.

[8] Brendel M, Focke C, Blume T, Peters F, Deussing M, Probst F (2017). Time Courses of Cortical Glucose Metabolism and microglial activity across the Life Span of Wild-Type mice: a PET study. J Nucl Med.

[9] Kello N, Anderson E, Diamond B (2019). Cognitive dysfunction in systemic lupus erythematosus: a case for initiating trials. Arthritis Rheumatol.

[10] Turpin S, Martineau P, Levasseur MA, Meijer I, Décarie JC, Barsalou J (2019). 18F-Flurodeoxyglucose positron emission tomography with computed tomography (FDG PET/CT) findings in children with encephalitis and comparison to conventional imaging. Eur J Nucl Med Mol Imaging.

[11] Bordonne M, Chawki MB, Doyen M, Kas A, Guedj E, Tyvaert L (2021). Brain ^18^F-FDG PET for the diagnosis of autoimmune encephalitis: a systematic review and a meta-analysis. Eur J Nucl Med Mol Imaging.

[12] Yin Y, Wu J, Wu S, Chen S, Cheng W, Li L (2022). Usefulness of brain FDG PET/CT imaging in pediatric patients with suspected autoimmune encephalitis from a prospective study. Eur J Nucl Med Mol Imaging.

[13] Komatsu N, Kodama K, Yamanouchi N, Okada S, Noda S, Nawata Y (1999). Decreased regional cerebral metabolic rate for glucose in systemic lupus erythematosus patients with psychiatric symptoms. Eur Neurol.

[14] Weiner SM, Otte A, Schumacher M, Klein R, Gutfleisch J, Brink I (2000). Diagnosis and monitoring of central nervous system involvement in systemic lupus erythematosus: value of F-18 fluorodeoxyglucose PET. Ann Rheum Dis.

[15] Lee SW, Park MC, Lee SK, Park YB (2012). The efficacy of brain (18)F-fluorodeoxyglucose positron emission tomography in neuropsychiatric lupus patients with normal brain magnetic resonance imaging findings. Lupus.

[16] Curiel R, Akin EA, Beaulieu G, DePalma L, Hashefi M (2011). PET/CT imaging in systemic lupus erythematosus. Ann N Y Acad Sci.

[17] Jorgensen A, Law I, Nielsen S, Jorgensen MB (2012). Fluorodeoxyglucose positron emission tomography in juvenile systemic lupus erythematosus with psychiatric manifestations: relation to psychopathology and treatment response in two cases. Rheumatology.

[18] Soubrier C, Faucher B, Guedj E, Kaphan E, Ebbo M, De Sainte Mari B (2020). Brain 18F-fluorodeoxyglucose positron emission tomography/computed tomography detection of neuropsychiatric lupus with normal cerebral magnetic resonance imaging. Rheumatology.

[19] Archambaud F, Bouilleret V, Hertz-Pannier L, Chaumet-Riffaud P, Rodrigo S, Dulac O (2013). Optimizing statistical parametric mapping analysis of 18F-FDG PET in children. EJNMMI Res.

[20] Tzourio-Mazoyer N, Landeau B, Papathanassiou D, Crivello F, Étard O, Delcroix N (2002). Automated anatomical labeling of activations in SPM using a macroscopic anatomical parcellation of the MNI MRI single-subject brain. NeuroImage.

[21] Mazzuca M, Jambaque I, Hertz-Pannier L, Bouilleret V, Archambaud F, Caviness V (2011). 18F-FDG PET reveals frontotemporal dysfunction in children with fever-induced refractory epileptic encephalopathy. J Nucl Med.

[22] Trotta N, Archambaud F, Goldman S, Baete K, Van Laere K, Wens V (2016). Functional integration changes in regional brain glucose metabolism from childhood to adulthood. Hum Brain Mapp.

[23] Ligot N, Archambaud F, Trotta N, Goldman S, Van Bogaert P, Chiron C (2014). Default mode network hypometabolism in epileptic encephalopathies with CSWS. Epilepsy Res.

[24] Krakauer M, Law I (2009). FDG PET brain imaging in neuropsychiatric systemic lupus erythematosis with choreic symptoms. Clin Nucl Med.

[25] Mackay M, Tang CC, Volpe BT, Aranow C, Mattis PJ, Korff RA (2015). Brain metabolism and autoantibody titres predict functional impairment in systemic lupus erythematosus. Lupus Sci Med.

[26] Mackay M, Vo A, Tang CC, Small M, Anderson EW, Ploran EJ (2019). Metabolic and microstructural alterations in the SLE brain correlate with cognitive impairment. JCI Insight.

[27] Van Bogaert P, Wikler D, Damhaut P, Szliwowski HB, Goldman S (1998). Regional changes in glucose metabolism during brain development from the age of 6 years. NeuroImage.

[28] Chételat G, Arbizu J, Barthel H, Garibotto V, Law I, Morbelli S (2020). Amyloid-PET and ^18^F-FDG-PET in the diagnostic investigation of Alzheimer’s disease and other dementias. Lancet Neurol.

[29] Pardini M, Huey ED, Spina S, Kreisl WC, Morbelli S, Wassermann EM (2019). FDG-PET patterns associated with underlying pathology in corticobasal syndrome. Neurology.

[30] Pilotto A, Premi E, Paola Caminiti S, Presotto L, Turrone R, Alberici A (2018). Single-subject SPM FDG-PET patterns predict risk of dementia progression in Parkinson disease. Neurology.

[31] Iaccarino L, Sala A, Caminiti SP, Santangelo R, Iannaccone S, Magnani G (2018). The brain metabolic signature of visual hallucinations in dementia with Lewy bodies. Cortex.

[32] Nishio Y, Yokoi K, Uchiyama M, Mamiya Y, Watanabe H, Gang M (2017). Deconstructing psychosis and misperception symptoms in Parkinson’s disease. J Neurol Neurosurg Psychiatry.

[33] Morningstar M, Hung A, Mattson WI, Gedela S, Ostendorf AP, Nelson EE (2020). Internalizing symptoms in intractable pediatric epilepsy: structural and functional brain correlates. Epilepsy Behav.

[34] Huang YS, Liu FY, Lin CY, Hsiao IT, Guilleminault C (2016). Brain imaging and cognition in young narcoleptic patients. Sleep Med.

[35] Braun AR, Randolph C, Stoetter B, Mohr E, Cox C, Vladar K (1995). The functional neuroanatomy of Tourette’s syndrome: an FDG-PET study. II: relationships between regional cerebral metabolism and associated behavioral and cognitive features of the illness. Neuropsychopharmacology.

[36] Shine JM, Lewis LD, Garrett DD, Hwang K (2023). The impact of the human thalamus on brain-wide information processing. Nat Rev Neurosci.

