# Supplementary figures and images for: Brain 18 F-FDG PET reveals cortico-subcortical hypermetabolic dysfunction in juvenile neuropsychiatric systemic lupus erythematosus
Source: EJNMMI Res. 2024 Apr 2;14:34. doi: 10.1186/s13550-024-01088-4 (PMC10987444; doi:10.1186/s13550-024-01088-4)

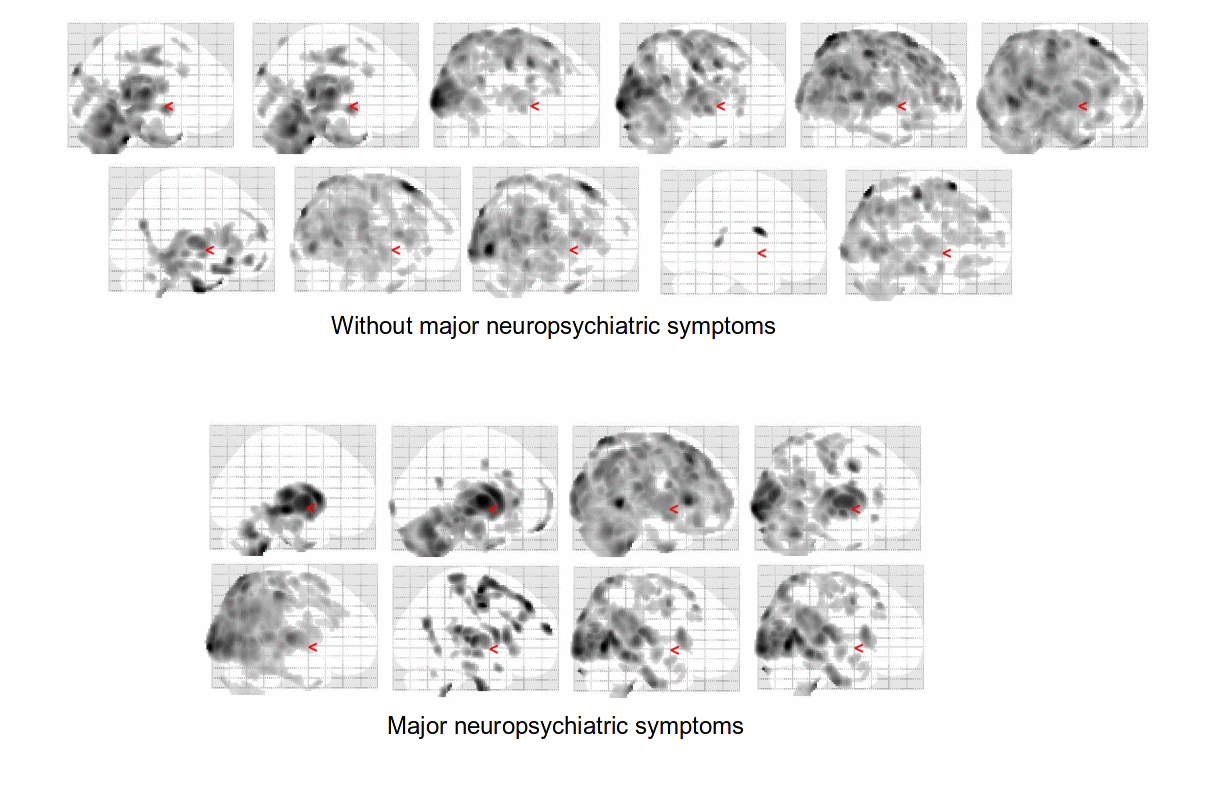

Supplement: Supplementary file 1 — Supplementary Material 1 [file 13550_2024_1088_MOESM1_ESM.gif]
